# Characterization of influenza infection in a high-income urban setting in Nairobi, Kenya

**DOI:** 10.1186/s41182-022-00463-y

**Published:** 2022-09-16

**Authors:** Gabriel Miring’u, Betty Muriithi, Hisashi Shoji, Samwel M. L. Symekher, Ernest Apondi Wandera, Claire Majisu, Mitsuo Takei, Koome Mwiraria, Yukie Saito, Satoshi Kaneko, Issei Tokimatsu

**Affiliations:** 1 Institute of Tropical Medicine, Nagasaki University-Kenya Medical Research Institute Project, 19993-00202, Nairobi, Kenya; 2 Embassy of Japan, La Paz, Bolivia; 3 Center for Virus Research, KEMRI, Nairobi, Kenya; 4 Lavington Pediatrics, Lavington, Nairobi, Kenya; 5 Forest Japan Medical Centre, Nairobi, Kenya; 6 J.G. Medical Clinic, Nairobi, Kenya; 7 Division of Clinical Infectious Diseases, Department of Internal Medicine, School of Medicine, Showa University, Tokyo, Japan

**Keywords:** Influenza virus, Prevalence, Diagnosis

## Abstract

**Background:**

Influenza viruses are an important cause of respiratory infections across all age groups. Information on occurrence and magnitude of influenza virus infections in different populations in Kenya however remains scanty, compromising estimation of influenza disease burden. This study examined influenza infection in an urban high-income setting in Nairobi to establish its prevalence and activity of influenza viruses, and evaluated diagnostic performance of a rapid influenza diagnostic test.

**Methodology:**

A cross-sectional hospital-based study was conducted in six private health facilities located within high-income residential areas in Nairobi from January 2019 to July 2020. Patients of all ages presenting with influenza-like illness (ILI) were recruited into the study. Detection of influenza virus was conducted using rapid diagnosis and reverse transcription–polymerase chain reaction (RT–PCR). Data were summarized using descriptive statistics and tests of association. Sensitivity, specificity and area under receiver operating characteristics curve was calculated to establish diagnostic accuracy of the rapid diagnosis test.

**Results:**

The study recruited 125 participants with signs and symptoms of ILI, of whom 21 (16.8%) were positive for influenza viruses. Of all the influenza-positive cases, 17 (81.0%) were influenza type A of which 70.6% were pandemic H1N1 (A/H1N1 2009). Highest detection was observed among children aged 5–10 years. Influenza virus mostly circulated during the second half of the year, and fever, general fatigue and muscular and joint pain were significantly observed among participants with influenza virus. Sensitivity and specificity of the diagnostic test was 95% (95% confidence interval 75.1–99.9) and 100% (95% confidence interval 96.5–100.0), respectively.

**Conclusions:**

Findings of this study shows continuous but variable activity of influenza virus throughout the year in this population, with substantial disease burden. The findings highlight the need for continuous epidemiologic surveillance including genetic surveillance to monitor activity and generate data to inform vaccine introduction or development, and other interventions.

## Introduction

Influenza viruses cause significant morbidity and mortality globally [18]. They result to annual epidemics that cause 3 to 5 million cases of severe disease leading to about 290,000–650,000-related deaths [33]. Despite availability of interventions, countries that are most likely to have a high burden of influenza infection are yet to take up influenza prevention and mitigation strategies especially vaccination [36]. Among the hinderances to adoption of core interventions is absence of data on influenza disease burden in various risk groups, that makes it difficult to evaluate usefulness of interventions or justify their uptake.

Importance of influenza viruses is derived from their capacity to cause pandemics or epidemics of varying intensity. The 2009 H1N1 pandemic clearly demonstrated the unpredictability of influenza virus evolution and the need for continuous monitoring for emerging strains. Presently, although H1, H2 and H3 are the main subtypes with greatest potential to cause pandemics [14], recent sporadic outbreaks of H5, H7 and H9 subtypes have raised serious concerns over public health threats posed by influenza viruses [16, 28]. In addition, diagnosis of influenza virus is challenging, because it is signs and symptoms overlap with those of other respiratory infections. Diagnosis is further limited by absence of efficient diagnostic tools particularly in clinical settings.

Recent studies have reported a high prevalence of influenza infection in sub-Saharan Africa, Asia–Pacific region and South America [18, 34]. Influenza is gaining importance in the tropical regions too, due to endemicity of influenza viruses in natural hosts [16], making these regions potential locales for emergence of novel strains. Furthermore, tropical regions play an important role in global influenza transmission [29], due to inherent factors that favor rapid transmission of influenza viruses. True burden of influenza in these regions, however, remains unknown, due to unpredictability of epidemics and limited capacity to generate local data on influenza virus activity coupled with competing health priorities.

Kenya set up a national sentinel surveillance system for influenza in 2006 in response to the avian Influenza A (H5N1) threat to monitor influenza virus activity and establish pandemic preparedness [20]. The system operates in six public county referral hospitals and the national referral hospital, that serve middle-to-low-income earners, largely excluding high-income earners. Although the system has enabled timely monitoring of influenza morbidity, generated data are likely to underestimate actual influenza virus burden in the public, since it lacks contribution from all key populations.

High income societies have a high likelihood of both local and international travel with a higher capacity for social mixing yielding a higher risk of transmission to and from their contacts. It is, therefore, necessary to understand the activity influenza virus in this population, and its contribution to national prevalence. Moreover, it has been postulated that travel and social contact bear a higher risk for transmission of influenza viruses [2, 11], with potential of introduction of novel strains.

This study examined influenza infections within high-income settings in Nairobi to establish disease prevalence and activity of influenza viruses within this population. Accuracy of the rapid diagnostic kit used in this study was also evaluated to establish its diagnostic performance in Kenyan settings.

## Methodology

### Study site

The study was conducted in Lavington, Upper hill, Riverside and Parklands areas in Nairobi County. These are well-established Nairobi suburbs that host commercial spaces, Embassies and high-end residences. They are mostly inhabited by diplomats, expatriates and local business owners, and are less populous compared to other areas of Nairobi. These areas were purposively selected, because the largest proportion of high-income earners in Nairobi, targeted by this study reside there. Furthermore, these areas are not covered by the sentinel influenza surveillance system, because none of the areas hosts a level four public health facility. Six health facilities located in these areas were selected to participate in the study. The health facilities were private primary health care facilities offering general physician out-patient services, although one had a specialized pediatric center and another offered additional specialized services. The criteria for inclusion of these facilities was availability of general practitioner services and capacity of the health facility. Big health facilities; in terms of ability to serve more people and with diagnostic services, or the most preferred in the area, where such information was available were selected to participate. These criteria served to control age bias, since general practitioner clinics serve patients of all ages, with the large health facilities allowing for inclusion of more participants. Most health facilities in the study areas are small to medium health facilities serving middle-level earners who live in the periphery of the suburbs or specialized clinics with a broad range of clientele. The health facilities that participated in this study were, therefore, the major health facilities in the study areas providing general physician services, whose clientele was the high-income earners and that served a large population of our target population.

### Study design and target population

This was a cross-sectional, hospital-based study. It was conducted from January 2019 to July 2020. The target population was high-income earners residing in wealthy suburbs in Nairobi. To establish actual influenza morbidity and its seasonal distribution, sample size was not pre-determined. All persons presenting with ILI who met the inclusion criteria were included in the study. Participants were selected using consecutive sampling. ILI was defined as Fever (> 37.5 °C), symptoms of upper airway inflammation (cough, nasal discharge, nasal congestion, sore throat and sputum, dyspnea), malaise and myalgia [32]. To be included in the study, patients with ILI were required to be presenting with at least one systemic symptom (fever, malaise and myalgia) and at least one symptom of upper airway inflammation, and to provide a written informed consent. Sampling was ended following depletion of funding coupled with substantial decline in patient volume, that was tentatively an effect of measures put in place to manage the COVID-19 pandemic.

### Study procedure

Patients were recruited during routine outpatient care. Upon providing consent, a pathological form was filled, detailing the participant’s demographic characteristics, vaccination, exposure history, signs and symptoms and treatment history. Occurrence of ILI during the year was recorded based on seasonal weather changes in Nairobi. Two nasopharyngeal swabs were collected thereafter from patients using standard procedure. Swabs were used for point-of-care diagnosis and molecular diagnosis. The swab intended for molecular diagnosis was collected in a virus transport media, stored at 4 °C–8 °C and transported to the NUITM Project Laboratory within 48 h for processing.

### Rapid detection of influenza

Rapid testing for influenza was done at the point of care using QuickNavi-Flu2 rapid test kit (DENKA SEIKEN CO., Ltd, Japan) for detection of Flu A and B. A nasopharyngeal swab was eluted in a pre-packaged buffer tube from which two drops were poured onto the sample panel. Time to reading of result was 3 to 8 min.

### Laboratory methods

Rapid test results were confirmed by convectional RT–PCR. Flu A (sub-types H1N1and H3N2) and Flu B (Victoria and Yamagata lineages) were detected using WHO validated protocols [32]. Matrix protein was targeted for detection of influenza A, using primer sets M30F2/08 (ATGAGYCTTYTAACCGAGGTCGAAACG) and M264R3/08 (TGGACAAANCGTCTACGCTGCAG). Sub-typing for determination of H1N1 (2009) and was based on detection of HA gene fragment, and HA and NA for H3N2. Primers HKU–SWF and HKU–SWR (sequences GAGCTCAGTGTCATCATTTGAA and TGCTGAGCTTTGGGTATGAA) were used for detection of H1N1 and H3A1F3, HARUc (H3), N2F387, NARUc (N2) for detection of H3N2 (Table 1). Detection of Flu B using HA gene fragments specific for each lineage failed to effectively detect Flu B cases that were positive by rapid test kit. A universal primer (BHA1-N and BHA1-C, Table 1) for Flu B targeting HA gene was, therefore, used to screen all samples for Flu B. Resulting positives were confirmed using real-time RT–PCR.

**Table 1:** PCR primers used in the study

Primer	Gene	Sequence	Influenza	Amplicon size
M30F2/08 M264R3/08	Matrix(M)	ATGAGYCTTYTAACCGAGGTCGAAACG TGGACAAANCGTCTACGCTGCAG	A (Universal)	244
HKU–SWF HKU–SWR	HA	GAGCTCAGTGTCATCATTTGAA TGCTGAGCTTTGGGTATGAA	A (H1NI1)	173
H3A1F3 HARUc	HA-3′ (H3)	TGCATCACTCCAAATGGAAGCATT ATATCGTCTCGTATTAGTAGAAACAAGGGTGTTTT	A (H3)	863
N2F387 NARUc	NA-3′(N2)	CATGCGATCCTGACAAGTGTTATC ATATGGTCTCGTATTAGTAGAAACAAGGAGTTTTTT	N2	1082
Bvf224 Bvr507	HA	ACATACCCTCGGCAAGAGTTTC TGCTGTTTTGTTGTTGTCGTTTT	B (Victoria)	284
BYf226 BYr613	HA	ACACCTTCTGCGAAAGCTTCA CATAGAGGTTCTTCATTTGGGTTT	B(Yamagata)	388
BHA1-N BHA1-C	HA	5'-AATATCCACAAAATGAAGGC 5'-AGCAATAGCTCCGAAGAAAC	Universal B	1119

RNA was extracted using ISOGEN-LS kit (Nippon gene, code No, 311-02621) following manufacturer’s instructions. Amplification was performed with QIAGEN One-step RT–PCR Kit (cat No; 210215 Qiagen, Valencia, CA, USA). Reaction mixture was amplified according to protocol by the World Health Organization, London Influenza Reference Center [32], except for universal primer for Flu B whose amplification condition was initial transcription at 50 °C for 30 min, and at 95 °C for 15 min, initial denaturation at 94 °C for 30 s, annealing at 58 °C for 30 s, extension at 72 °C for 80 s for 40 cycles, finally final extension for 10 min.

### Gel electrophoresis

PCR product was electrophoresed using Mupid-exU tanks, 8 µl of PCR products, mixed with 2 µl of loading buffer (6 × Nippon gene) was electrophoresed in a 1.5% agarose gel in a 1X TAE running buffer, at 100 V for 30–35 min, using Nippon gene ladder (0.1–2 kb).

The gel was stained in ethidium bromide solution for 10 min, then visualized through UV trans-illuminator imaging system (E-Box-1000/26 m).

### Data analysis

Data were analyzed using STATA 14. Descriptive statistics were used to calculate proportions. Chi-square test or fisher’s exact test was used to test for association between detection of influenza virus and characteristics of participants or signs and symptoms. Diagnostic test results from the rapid test kit were compared with RT–PCR test results to establish diagnostic performance of the test kit. Sensitivity, specificity and area under receiver operating characteristics (ROC) curve were calculated to establish accuracy of the test kit.

## Results

Between January 2019 and July 2020, 125 patients with ILI were recruited into the study. Most of the participants (54.4%) were female. Age of participants ranged from 5 months to 78 years with most participants; 26.4% aged below 5 years. Most of the participants; 45.8% were of Asian ethnicity. Over half of the participants were not vaccinated, as shown in Table 2. Of the participants who had travelled within 2 weeks preceding presentation for care, 14.3% tested positive for influenza infection. Seasonal distribution of influenza infection was based on data collected in 2019, since samples were collected throughout the year, to avoid overestimating disease burden for the seasons including months during which samples were collected in 2020, and underestimating burden in the seasons including months after which sample collection had stopped. Infections peaked from October to November. Age group 5–10 years, and March to May and October to November seasons were significantly associated with detection of influenza virus.

**Table 2:** Characteristics of study participants and rate of influenza infection

Characteristic	Influenza virus positive (%) (*n* = 21)	Influenza virus negative (%) (*n* = 104)	Total population (%)	*p* value*
Gender				
Male	10 (17.5)	47 (82.5)	57 (45.6)	0.839
Female	11(16.2)	57 (83.2)	68 (54.4)	0.839
Age (years)				
0–4	3 (9.1)	30 (90.9)	33 (26.4)	0.132
5–10	10 (40.0)	15 (60.0)	25 (20.0)	0.001
11–19	2 (15.4)	11 (84.6)	13 (10.4)	0.623
20–34	0	9 (100.0)	9 (7.2)	0.180
34–49	4 (12.9)	27 (87.1)	31 (24.8)	0.354
50 years and above	2 (14.3)	12 (85.7)	14 (11.2)	0.571
Ethnicity				
African	6 (20.7)	23 (79.3)	29 (23.2)	0.523
Asian	9 (16.4)	46 (83.6)	55 (44.0)	0.908
American/European	6 (18.2)	27 (81.8)	33 (26.4)	0.805
Unknown	0	8 (100.0)	8 (6.4)	0.219
Vaccination status				
Vaccinated	2 (11.8)	15 (88.2)	17 (13.6)	0.425
Ever been vaccinated	0	11 (100.0)	11 (8.8)	0.120
Never been vaccinated	19 (20.2)	75 (79.8)	94 (75.2)	0.060
Unknown	0	3 (100.0)	3 (2.4)	0.573
Travel history				
Travel history	8 (13.3)	52 (86.7)	60 (48.0)	0.319
Onset of symptoms within 14 days of travel	5 (14.3)	30 (80.7)	35 (58.3)	0.557
Onset of symptoms beyond 14 days of travel	3 (12.0)	22 (88.0)	25 (41.7)	0.557
No travel history	13 (20.0)	52 (80.0)	65 (52.0)	0.319
Seasons (*n* = 101)				
March–May	0	25 (100.0)	25 (24.7)	0.006
June–September	3 (12.5)	32 (87.5)	24 (23.8)	0.389
October–November	12 (38.7)	19 (61.3)	31 (30.7)	< 0.001
December–February	2 (9.5)	19 (90.5)	21 (20.8)	0.266

^*^Seasonal distribution of ILI and influenza cases was based on seasonal weather changes in Nairobi

^*^p value of association between characteristic and detection of influenza virus

### Detection of influenza virus among the study participants

Influenza virus was detected in 16.8% (21/125) of the participants. Influenza A viruses were the most frequently detected (18/21, 81.0%, Table 3), with Influenza A/H1N1 being the predominant subtype (59.1%). Co-infection with influenza A and B was not detected. Children aged between 5 and 10 years had the highest rate of infection relative to other age groups and most of the infected participants had never been vaccinated (Table 2).

**Table 3:** Influenza virus types

Type	*n*	%
Influenza A	17	81.0
*Pandemic H1N1*	12	70.6
*A/H3N2*	3	17.6
*A/Not typeable*	2	11.8
Influenza B	4	19.0

### Clinical presentation among study participants

Clinical symptoms varied among the subjects. Nasal discharge/congestion (82.4%), general fatigue (76.0%), cough (64.8%) and muscular and joint pain (54.0%) were the most frequently elicited symptoms of ILI (Table 4). Fever, general fatigue and muscular and joint pains were significantly associated with influenza virus detection.

**Table 4:** Distribution of signs and symptoms and association with influenza

Sign/symptom	No. of participants with sign or symptom (%)	Influenza positive participants with sign or symptom (%)	*p* value*
Fever	66 (52.8)	16 (24.2)	0.019
General fatigue	95 (76.0)	20 (20.1)	0.024
Muscular and joint pain	60 (54.0)	15 (25.0)	0.002
Cough	81 (64.8)	16 (19.7)	0.231
Nasal discharge/congestion	103 (82.4)	20 (19.4)	0.090
Sore throat	63 (50.4)	12 (19.1)	0.498

^*^p value of association between sign or symptom and detection of influenza virus

### Performance of the QuickNavi-Flu2 rapid test kit

**Fig. 1:**
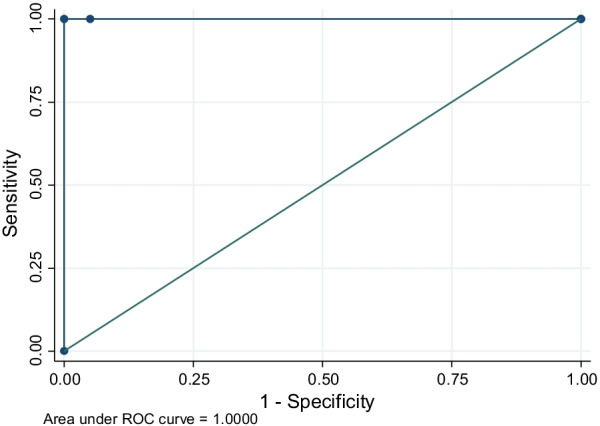
ROC curve for rapid diagnostic test for Influenza with convectional RT–PCR as the gold standard

Of the 21 samples that tested positive for influenza, one sample was not screened using the rapid test kit and was excluded in calculation of sensitivity and specificity. The rapid diagnostic kit detected 19 true positives and 1 false negative. Sensitivity of the rapid diagnostic kit was 95% (95% CI 75.1–99.9) and specificity was 100% (95% CI 96.5–100.0). The area under ROC curve was 1.0, as shown in Fig. 1.

## Discussion

Influenza virus infections remain an important cause of respiratory infections. In this study, 16.8% of the participants presenting with ILI were infected with the influenza virus. Observed prevalence was within the national estimate, that has been shown to range from 12% to 19% [9, 12, 20, 30] and up to 27% among medically attended patients [10]. Influenza A/pandemic H1N1 was the most prevalent subtype during the study duration, with minimal detection of H3N2 during the entire period. This subtype distribution was unlike that observed in the surveillance system during the same period, where H3N2 predominated during early 2019, being overtaken by H1N1 that predominated later in the year into 2020 [13]. Failure to observe predominance of H3N2 was possibly due to the study’s small sample size, or an interplay of factors including transmission dynamics and host susceptibility. Only 18% of the influenza cases were due to influenza B, which was contrary to its high activity in the national sentinel surveillance [13]. Prevalence of influenza virus in this population and its setting was, therefore, quite similar to that of the population covered by the sentinel surveillance, with modest but noticeable differences in subtype distribution.

Increased influenza activity was observed between October and November of 2019, with a few more detections between June and September of 2019 compared to other seasons. Although this study did not observe distinct seasonality of influenza infection, depicted trend was in agreement with data from the national sentinel surveillance that has shown that most influenza cases occur during the second half of the year, with July and October being the median onset months [9, 20]. Absence of influenza cases during the March to May rainy season equally agrees with reports that have showed that influenza activity at times decreases between April and May [9, 20, 30]. Studies in other tropical countries have similarly observed one or more peaks per year [15, 24] that have been shown to coincide with periods of higher humidity [6, 27], although variability of influenza seasonality in the tropical regions is widely acknowledged. The study further observed a significant association between occurrence of influenza and seasons, both low and intense circulation seasons, suggesting that climatic factor could be an important determinant for occurrence of influenza infection. Given current efforts to introduce an influenza vaccine in Kenya, these findings can be used to inform timing of vaccination campaigns.

Prevalence of influenza was highest among children aged 5–10 years, with substantial detection among persons aged 50 years and above. On the contrary, results from a systematic analysis of burden of influenza in Kenya using data collected before 2013 [10] and subsequent analysis of national sentinel surveillance data [20, 30] observed a high prevalence among children aged below 5 years, which are consistent with data from other regions [7, 25]. High prevalence among children aged 5–10 years may be related to high tendency of person-to-person contact especially within school or play settings, and hygiene behavior of this age group that affects pathogen density and host proximity, favoring viral transmission [8], coupled with the fact that children shed viruses in greater quantities and over longer durations. This age group, therefore, gets infected with influenza easily after which it initiates active transmission in the community and especially to vulnerable age groups. Consequently, prevalence in this age group may appear higher in some samples but it is certainly lower than that of children aged below 5 years in community settings. High incidence among persons aged 50 years and above could be attributed to underlying health conditions and weakened immunity with advancing age.

Only 13.6% of the participants had valid influenza vaccination, although 8.8% more reported that they had ever been vaccinated. Most of the vaccinated participants were of Asian and American/European ethnicities, despite the nature of the study’s setting, where usage of influenza vaccination is expected to be high among locals. Observed low coverage especially among locals could be due to low awareness and acceptance of the influenza vaccine. Kenya lacks an official influenza vaccination policy and there are no programs in place to promote usage of the vaccine. Utilization of influenza vaccine in Kenya is further complicated by its tropical orientation that requires utilization of both northern and southern vaccine formulations at particular periods.

Fever, general fatigue and muscular and joint pain were significantly associated with an influenza infection, although most patients with influenza also presented with cough and runny nose. Other studies have observed cough, fever, myalgia and weakness [23], cough and fever [5], and fever, cough and rhinorrhea [29] as significant predictors of influenza infection. Differences in predictive symptoms may be due to the generally diverse clinical presentation of influenza. In addition, signs and symptoms of influenza vary by age [3], country or region [19], and even by infecting subtype [4, 35], which hampers establishment of a definitive diagnosis. Clinical definition of influenza, therefore, is largely based on case definition for ILI, that is used to predict influenza activity. Application of ILI definition for clinical differentiation of influenza from other respiratory viruses that present through ILI remains difficult [19].

Sensitivity of Quick Navi-Flu2 in comparison with RT–PCR as the gold standard was lower than its specificity, although both were sufficiently high for effective diagnosis of influenza, considering factors that affect sensitivity of rapid influenza detection kits [22, 31]. Khamsorn et al. [21] and Akaishi et al. [1] similarly reported sensitivities ranging from 81.7% to 85.7% that were lower than 98.8–100% specificities for Quick Navi-Flu2 test kit against real time RT–PCR and viral isolation methods, respectively. Although point of care testing for influenza is not routine in most health care facilities in Kenya, such a test kit may have a role in efficient and sustainable management of influenza infections, especially in public health facilities, where diagnostic capacity is even lower.

This study had some limitations that could affect application of its findings. The study area was restricted to high-income residential areas which limited target population, since only small private practice health facilities operate in such areas. This impacted negatively on the sampling frame yielding a small sample size. The sample size was further affected by relative global reduction in the incidence of influenza in 2020 following outbreak of COVID-19 [17, 26]. The epidemic changed social and health behaviors resulting to reduction of influenza cases. The study also covered high-income residential areas in Nairobi only, although it is the largest high-income area in Kenya. There could be variation in influenza activity in other related settings that could not be captured in this study. Moreover, genetic characterization of detected viruses was not explored limiting its contribution to information on virus evolution.

## Conclusions

This study expands data on influenza disease epidemiology in Kenya, including disease burden, and strain distribution. Influenza virus epidemiology in this study was similar to that reported by the sentinel surveillance, although strain distribution differed slightly. Further research on influenza virus transmission dynamics in this population is critical, to understand its role in overall influenza burden in Kenya. Higher infection rate observed among older children is this study highlights this age group as a priority target for influenza control programs, since it is not included in vaccination programs yet it has potential to drive transmission in the community. Diagnostic capacity of QuickNavi-Flu2 rapid test kit was confirmed in this study too, with high sensitivity and specificity.

## Data Availability

Data and materials used to conduct this study are available on request.
